# First 1000 Days Strategies to Prevent Childhood Obesity: A Narrative Review and Recommendations From the EndObesity Consortium

**DOI:** 10.1111/ijpo.70060

**Published:** 2025-10-17

**Authors:** Mireille C. Schipper, Anna Manshanden, Kaat Philippe, Natàlia Ferré, Veronica Luque, Marion Lecorguillé, Adrien M. Aubert, Kathrin Guerlich, Camille Le Gal, Shweta Feher, Ester Parada‐Ricart, Joaquin Escribano, Veit Grote, Berthold Koletzko, Teresa Primavesi‐Poggio, Katharina Reiss, Jaap C. Seidell, Sandrine Lioret, Catherine M. Phillips, Barbara Heude, Romy Gaillard

**Affiliations:** ^1^ The Generation R Study Group, Erasmus MC University Medical Center Rotterdam The Netherlands; ^2^ Department of Pediatrics, Sophia's Children's Hospital, Erasmus MC University Medical Center Rotterdam The Netherlands; ^3^ Department of Health Sciences, Faculty of Science Vrije Universiteit Amsterdam Amsterdam The Netherlands; ^4^ School of Public Health, Physiotherapy and Sports Science University College Dublin Dublin Ireland; ^5^ Pediatrics Nutrition and Development Research Unit Institut d'Investigació Sanitària Pere Virgili Reus Spain; ^6^ Universitat Rovira i Virgili Reus Spain; ^7^ Université Paris Cité and Université Sorbonne Paris Nord Inserm, INRAE, Center for Research in Epidemiology and StatisticS (CRESS) Paris France; ^8^ Division of Metabolic and Nutritional Medicine, Department of Pediatrics, Dr. von Hauner Children's Hospital LMU University Hospital, LMU Munich Munich Germany; ^9^ German Center for Child and Adolescent Health Munich Germany; ^10^ Child Health Foundation – Stiftung Kindergesundheit c/o Dr. von Hauner Children's Hospital Munich Germany; ^11^ European Foundation for the Care of Newborn Infants Munich Germany; ^12^ Healthy Start Network Federal Office for Agriculture and Food Bonn Germany

**Keywords:** childhood obesity, european consortium, first 1000 days of life, lifestyle, prediction, prevention

## Abstract

Childhood obesity remains a major global public health challenge, leading to significant short‐ and long‐term adverse health outcomes and imposing substantial societal costs. Recognising the critical importance of early intervention, the Horizon2020 EU‐funded JPI Consortium EndObesity has prioritised the first 1000 days of life, from preconception to 2 years of age, as a key window for obesity prevention strategies. This narrative review synthesises findings from the EndObesity Consortium, summarising evidence from large multi‐cohort studies on the influence of family‐based health behaviours in the first 1000 days on offspring obesity risk, the potential of childhood obesity prediction models in the first 1000 days, and strategies to enhance prenatal and postnatal interventions to prevent childhood obesity development. Finally, we present recommendations for research, practice, and policy to address the complex, multifaceted challenges of childhood obesity prevention in the first 1000 days.

## 1 Introduction

The World Obesity Atlas 2024 estimates that by 2035 more than 750 million children aged 5–19 years will be living with overweight or obesity [1]. Childhood overweight and obesity are a major public health concern, leading to significant short‐term and long‐term adverse health outcomes and placing a substantial burden on quality of life and societal costs [2, 3]. These consequences can be severe and long‐lasting, as childhood obesity has been associated with increased rates of adult obesity, cardiovascular diseases, and even premature mortality from endogenous causes in early adulthood [4].

Accumulating evidence suggests that developmental adaptations in early life, in response to an adverse in utero and early‐childhood environment, increase susceptibility to obesity later in life [5]. Modifiable adverse family‐based lifestyle factors during preconception, pregnancy, and early childhood, collectively covering the first 1000 days of life (from preconception to 2 years of age), are highly prevalent and may contribute to an adverse early‐life environment, increasing the risk of childhood obesity [5, 6, 7]. These modifiable adverse family‐based lifestyle factors include health‐related behaviours such as diet, physical activity, sleep, stress management, and substance use. Adverse lifestyle factors often cluster within families and are more common among families with a low socio‐economic position or from ethnic minority groups [8, 9]. The transition to parenthood is considered a time when parents‐to‐be are more receptive to guidance and presents a key opportunity to address these family‐based adverse lifestyle factors to improve the health of the unborn child [10]. Thus, the first 1000 days represent a unique window of opportunity for early‐life prevention of childhood obesity and the reduction of social inequality in health across the life course. The tremendous potential for novel prevention strategies from the start of life onwards for childhood obesity prevention has yet to be capitalised on. The current scarcity of translation of research evidence into effective public health and clinical prevention strategies may stem from our limited understanding of family‐based health behaviour patterns during the first 1000 days related to childhood obesity development, poor available early‐life childhood obesity prediction tools, and challenges in developing, implementing, and evaluating childhood obesity prevention strategies [11, 12, 13, 14, 15].

**FIGURE 1:**
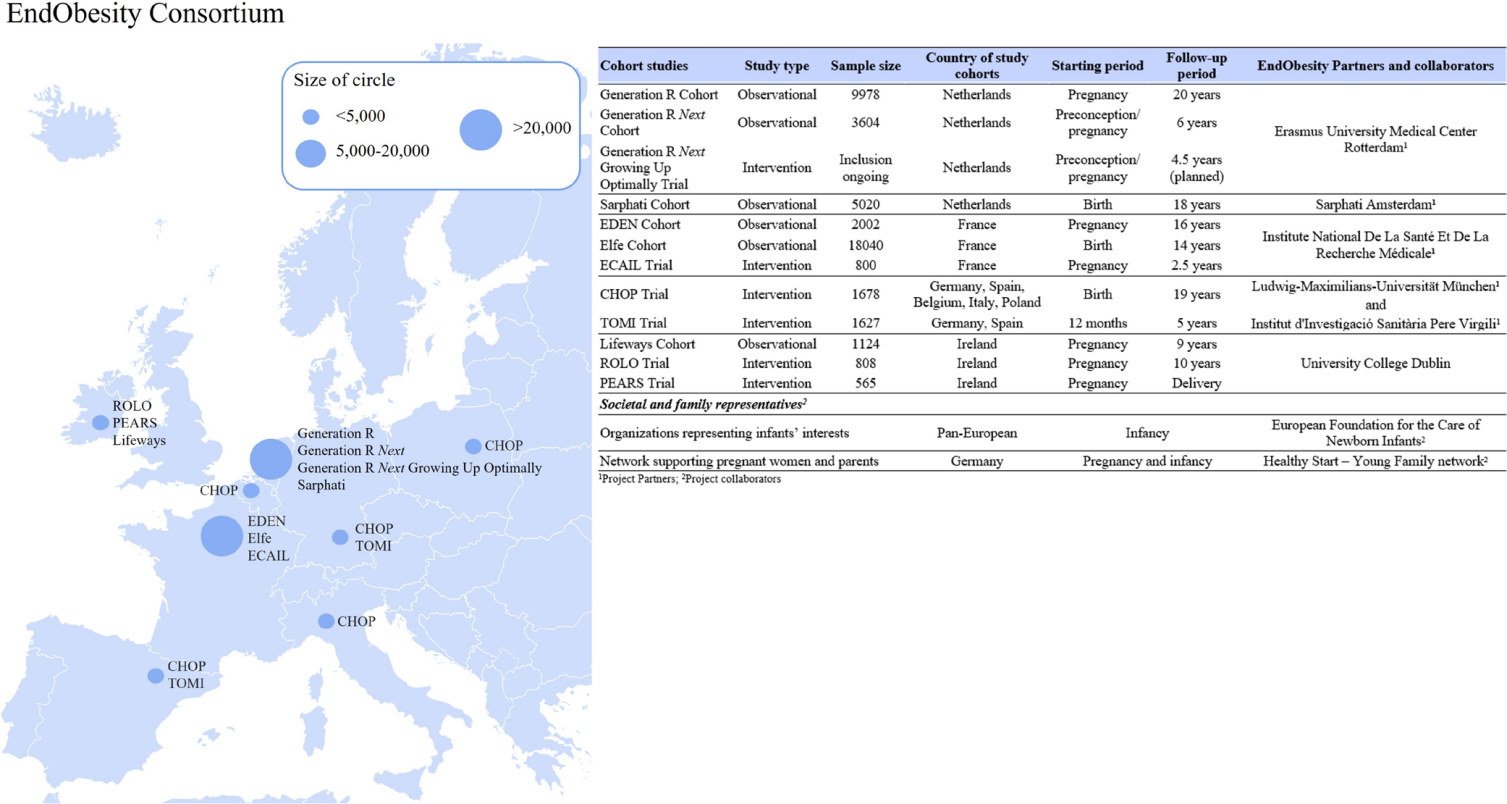
Overview of the EndObesity Consortium partners and collaborators. Circles on the map represent countries involved and approximate sample sizes of the contributing studies. The accompanying table provides details on each partner institution, partner‐specific studies and collaborators.

**FIGURE 2:**
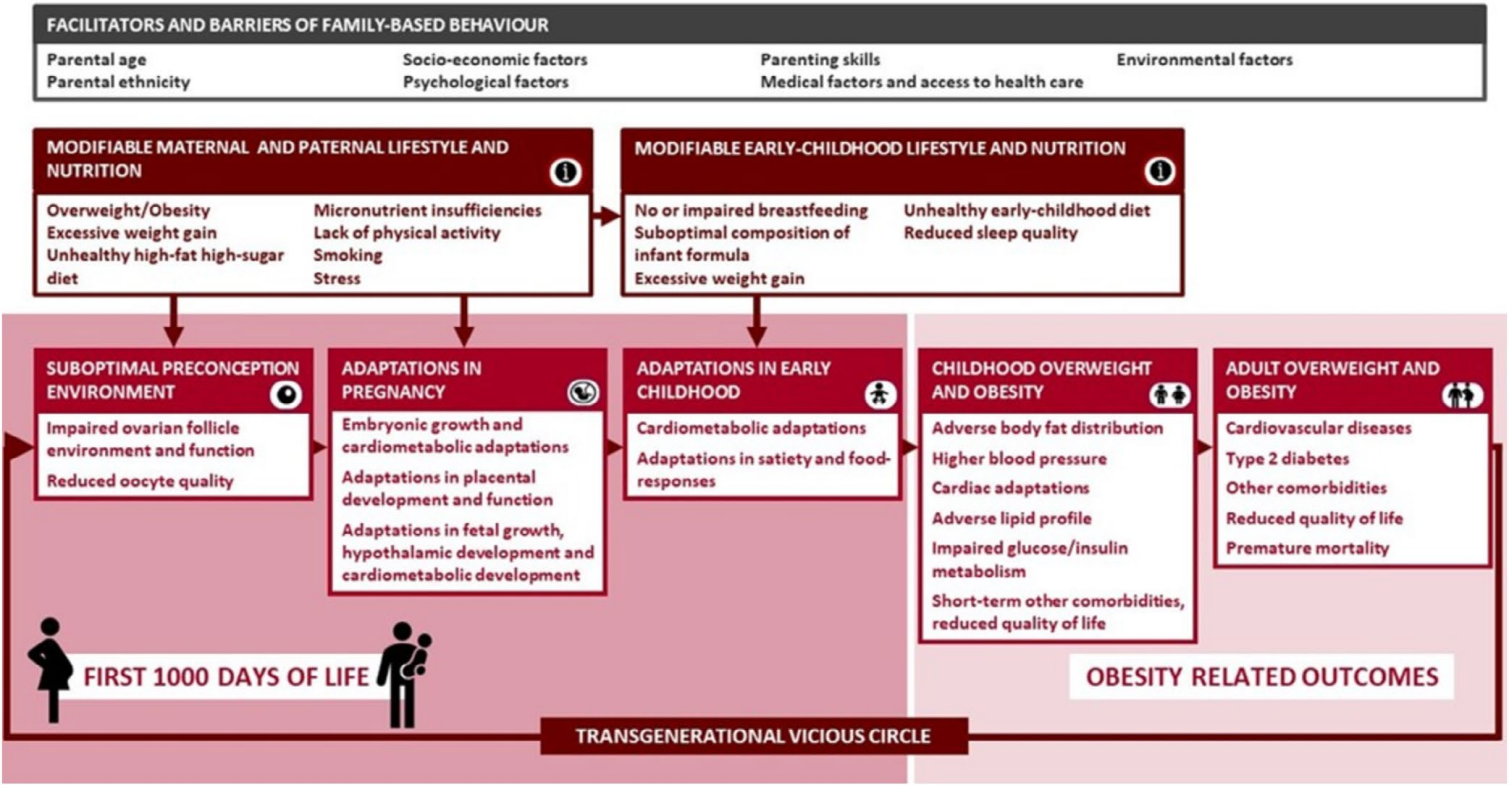
Conceptual framework for the EndObesity Consortium. i represents family‐based adverse modifiable lifestyle factors in preconception, pregnancy and early‐childhood related to increased risks of childhood obesity via independent effects and synergistic interacting effects. These factors urgently need to be used for prediction of childhood obesity and be the target of intervention strategies in these crucial transition periods. Maternal lifestyle and nutrition directly influence the preconception and pregnancy environment important for the oocyte and developing foetus. Paternal lifestyle influences maternal and family‐based lifestyle, highlighting the importance of targeting both parents. Facilitators and barriers may influence these family‐based health behaviours, behavioural change and obesity risk. These factors may also be used for prediction of childhood obesity and need to be considered in prevention and implementation strategies.

To address these gaps, we established a unique European collaboration, the EndObesity Consortium, which includes six multi‐disciplinary, complementary European research partners conducting 12 ongoing observational and intervention studies from preconception until young adulthood, two parent–childhood organisations, and a diverse set of national stakeholders (Figure 1). Through this collaboration, we brought together a team of European researchers, stakeholders, and consumer‐representative organisations with strong multidisciplinary expertise on the development of childhood obesity and a wide range of comorbidities, nutrition, lifestyle, social and behavioural sciences, implementation sciences, life course epidemiology, clinical trials, guideline development, and stakeholder research partnership. The overall objective of the EndObesity Consortium was to identify novel insights for innovative, multidisciplinary strategies for the prevention of childhood obesity by targeting adverse family‐based lifestyle factors in three crucial transition periods: the preconception period, pregnancy, and early childhood, together covering the full first 1000 days of life. Although improving family‐based lifestyle factors in one crucial transition period may already reduce childhood obesity risk, targeting all three crucial transition periods is likely to lead to synergistic effects for reducing childhood obesity risk. The conceptual framework, which forms the overall hypothesis for this consortium, is shown in Figure 2. To build a relevant and credible evidence base for novel insights into childhood obesity prevention, EndObesity combined data from high‐quality, multi‐ethnic cohorts and intervention trials across diverse European countries, with a focus on critical developmental periods and long‐term longitudinal follow‐up. A key strength was the collaboration with the EU Child Cohort Network, enabling joint analyses of already harmonised data between cohort studies and trials, supporting long‐term sustainability of this collaboration [16]. The EndObesity Consortium used a structured, multi‐method approach including study‐specific and multi‐study data analyses from observational and intervention studies, systematic or narrative review approaches of available evidence, qualitative approaches including interviews with healthcare professionals and focus group meetings with parents as end users, and peer discussions and consensus discussions with the full consortium, parent–childhood organisations, and national stakeholders from several European countries.

This narrative review brings together the insights generated within the EndObesity Consortium to summarise current knowledge on how family‐based lifestyle factors in the first 1000 days influence the development of childhood obesity and to explore their potential public health impact. First, we review findings from large‐scale cohort‐specific and multi‐cohort observational studies on family‐based lifestyle factors in the first 1000 days, socio‐ecological determinants, and childhood obesity risk. Next, we explore opportunities for early‐life risk prediction and prevention of childhood obesity in the first 1000 days by reviewing findings from large‐scale observational and intervention studies. Finally, we identify key priorities for future research and provide recommendations for translating current evidence into clinical practice and policy based on large‐scale data analyses, qualitative studies with healthcare professionals and parents, and consensus discussions within the consortium. These include potential public health and clinical strategies to optimise family‐based lifestyle factors from the preconception period until early childhood, with the goal of breaking the intergenerational cycle of obesity and reducing social inequalities.

### 1.1 Family‐Based Lifestyle Factors in the First 1000 Days and Childhood Obesity Risk

Family‐based lifestyle factors play an important role in shaping childhood health from the earliest stages of life. Although the importance of the first 1000 days in childhood obesity development is increasingly recognised, most research has focused primarily on maternal influences during pregnancy, often overlooking the critical preconception period and potential independent and combined effects of paternal factors [2, 3, 4]. Rather than a single period that leads to programming of obesity in later life, the development of obesity seems to be the result of cumulative effects of developmental adaptations across the life course, starting in the earliest phases of life. For example, an individual participant data meta‐analysis from 37 pregnancy and birth cohort studies found that higher maternal pre‐pregnancy BMI and gestational weight gain were associated with an increased risk of overweight and obesity in children aged 2–18 years [17]. Similarly, findings from the Generation R Study showed that a higher maternal pre‐pregnancy BMI was associated with greater abdominal, pericardial, and liver fat accumulation at mid‐childhood (ages 6–10 years) [18, 19, 20]. More detailed analyses of repeated weight exposure data before and during each trimester of pregnancy suggest that especially maternal weight before and, to a lesser extent, during the first trimester are associated with adverse body fat outcomes in mid‐childhood [19, 20]. In addition, suboptimal maternal dietary intake and related glucose and lipid concentrations, and smoking during the first half of pregnancy have been associated with mid‐childhood obesity outcomes [21, 22, 23, 24]. Paternal lifestyle factors during the first 1000 days are often correlated with those of mothers and may significantly influence offspring health outcomes [25, 26, 27, 28]. Mainly in animal studies, paternal preconception BMI, diet, and smoking habits have been linked to epigenetic modifications during spermatogenesis and alterations in seminal plasma, which may have lasting consequences for embryonic and childhood development [29, 30]. Findings from the Generation R *Next* study, a population‐based prospective cohort study from preconception onwards, highlight the importance of parental nutrition and weight status even before conception. Higher maternal and paternal pre‐pregnancy BMI and lower paternal dietary carbohydrate quality were shown to be independently associated with reduced fertility and a higher risk of miscarriage, while paternal PUFA‐rich food consumption was associated with improved fertility [31, 32, 33]. Given that early‐life metabolic programming begins at conception, these paternal influences on fertility may have downstream effects on offspring development and obesity risk. Findings from the Irish Lifeways cross‐generational cohort study and the Generation R Study suggest that paternal BMI and dietary quality (assessed using the Dietary Approaches to Stop Hypertension [DASH] and dietary inflammatory index [DII]) influence post‐natal offspring adiposity risk [18, 34, 35]. However, when a combined measure such as a paternal lifestyle score was considered, a low score was associated with a greater waist‐to‐height ratio, but not with increased BMI in childhood, suggesting that underlying mechanisms or pathways linking paternal lifestyle and offspring adiposity remain unclear [36].

Within EndObesity, we conducted multi‐cohort analyses across four European birth cohorts using a harmonised data and analysis approach to identify parental lifestyle patterns in preconception and during pregnancy and their association with childhood obesity risk [37, 38]. Including both maternal and paternal factors in principal component analyses, we identified consistent lifestyle patterns across cohorts. Patterns characterised by high parental BMI, parental smoking, maternal low diet quality, or high sedentary behaviour before or during pregnancy were associated with a higher risk of overweight and obesity throughout childhood (5–12 years of age) [37]. To enhance the potential for translation, we developed a priori Healthy Lifestyle Score (HLS) during the first 1000 days and analysed their association with childhood weight status [38]. Three composite HLS were calculated: a maternal pregnancy HLS based on diet quality (DASH and DII score), physical activity, smoking, alcohol consumption during pregnancy, and pre‐pregnancy BMI; a parental pregnancy HLS that additionally considered paternal BMI, physical activity, and smoking during pregnancy; and an infancy HLS including breastfeeding duration, age of solid food introduction, and exposure to passive smoking. Few families exhibited an optimal lifestyle, with 3.4%–10.0% scoring the maximum for the maternal HLS, 1.9%–3.7% for the parental HLS, and 12.2%–23.6% for the infancy HLS [38]. Higher maternal and paternal HLS during pregnancy were consistently associated with a lower risk of offspring overweight and obesity throughout childhood (5–12 years of age), whereas associations between the infancy HLS and the risk of offspring overweight and obesity were less consistent [38].

Collectively, these findings reinforce the robustness of our results, demonstrating consistent associations between parental lifestyle factors in the first 1000 days and childhood overweight and obesity across different analytical approaches. However, it is important to recognise that these lifestyle behaviours are shaped by broader social contexts. Interest in these social determinants of health has grown, recognising their role in behaviour and health disparities [39, 40]. These ‘upstream’ factors differ from individual behaviours and traits (‘downstream’ influences) and include broader social, commercial, and structural influences [41]. Few studies have comprehensively explored socio‐ecological factors influencing parental lifestyle factors during the first 1000 days [42]. Within EndObesity, we addressed this gap by conducting hierarchical linear regression analyses on parental lifestyle pattern scores [43, 44]. We first examined the impact of parental socio‐economic and socio‐demographic characteristics, followed by the urban environment, and finally considered psychosocial factors and health‐care access [44]. Our findings showed that higher socio‐economic position, foreign geographical background, and wealthier neighbourhoods were associated with a healthier parental lifestyle during pregnancy. In contrast, multiparity and psychiatric disorders during pregnancy were linked to unhealthier parental lifestyle [44]. Recent findings from the French ELFE and Dutch Generation R cohort further demonstrated that the previously identified parental lifestyle patterns during pregnancy can partially explain the association of parental socio‐economic position with childhood overweight risk [45]. Recognising these potential facilitators and barriers is essential for developing tailored family‐based health interventions that integrate both individual behaviours and broader structural influences to more effectively prevent childhood obesity during the first 1000 days.

### 1.2 Early‐Life Prediction of Childhood Obesity

Early identification of children at risk and subsequent prevention is crucial due to the persistent nature of obesity which tracks from childhood into adulthood [4]. Over 60% of children with overweight or obesity remain living with overweight or obesity as adults, significantly increasing their risk for hypertension, type 2 diabetes, coronary artery disease, and premature mortality [4, 46, 47, 48]. Nevertheless, these adverse outcomes can be fully reversed in children who attain a healthier weight before adulthood, emphasising the importance of early intervention [4, 46, 47, 48, 49]. To facilitate early identification and prevention, knowledge about risk factors must be translated into prediction models and subsequent prediction tools for clinical and preventive care. These prediction tools enable early identification of individuals at high risk of childhood obesity, allowing for targeted prevention strategies from the earliest phase of life onwards, optimising body weight during childhood and improving health outcomes across the life course.

Advances in our understanding of early‐life determinants of childhood obesity have improved risk prediction and model development [50, 51, 52, 53, 54, 55, 56]. A systematic review identified eight prediction models for childhood overweight and obesity, with reported area under the receiver operating curve (AUROC) values ranging from 0.64 to 0.91 for model development and 0.75–0.91 for model internal validation, reflecting moderate to excellent predictive performance [55]. Despite their promising potential, these models face limitations that hinder their clinical utility. Most existing models have a static nature, assessing risk factors at a single point in time, such as infancy or early childhood, without capturing the dynamic changes in risk from preconception through early childhood. Additionally, these models typically predict obesity at a single age, overlooking the evolving nature of weight status across childhood [57]. Finally, while several models show moderate to good predictive performance, the majority have been developed using small sample sizes, and few have been externally validated [56]. To build on the strengths of existing models and address their limitations, EndObesity is currently developing and validating dynamic childhood obesity prediction models using risk factors in preconception, pregnancy/birth, and infancy. We aim to develop dynamic models that continuously update risk factors across the first 1000 days, allowing for a responsive and accurate assessment of obesity risk as the child develops. This dynamic monitoring enables healthcare professionals to track changes in risk estimates between visits, allowing for more timely and tailored interventions. Based on interviews with healthcare professionals on their needs for better childhood obesity prevention, we believe that dynamic prediction approaches represent a significant advancement and will offer a valuable future tool for healthcare professionals to more effectively tailor interventions.

### 1.3 Intervention Strategies in the First 1000 Days to Prevent Childhood Obesity

A variety of interventions during pregnancy have been evaluated in intervention studies to prevent or reduce modifiable factors associated with an increased risk for childhood obesity [58]. These interventions, often targeting maternal lifestyle factors such as diet and physical activity, have shown small positive effects on gestational weight gain and some birth outcomes, including lower rates of caesarean sections and preterm births [59]. However, systematic reviews provide limited evidence that pregnancy‐based lifestyle interventions effectively reduce childhood overweight and obesity [11, 60, 61]. For instance, Raab et al. reviewed 20 prenatal lifestyle trials involving 11 385 women and found no significant impact on weight, length, BMI, or corresponding *Z*‐scores in children aged 1 month to 7 years [61]. A narrative systematic review, including 24 interventions from 33 articles, demonstrated that interventions during pregnancy and up to 2 years of age specifically tailored to socio‐economically disadvantaged families were more effective, showing improvements in behavioural and anthropometric outcomes in young children [62]. Current literature emphasises the importance of preconception health, advocating for interventions starting before pregnancy [7, 63]. Despite this, evidence on the effectiveness of preconception interventions is extremely sparse, with only a few studies reporting child outcomes [64, 65]. To explore why preconception and pregnancy interventions have had limited success in reducing childhood overweight and obesity, a recent scoping review within EndObesity mapped complex intervention and process evaluation components and consulted a team of experts [66]. This scoping review included 40 publications corresponding to 27 trials involving 14 319 women. This review identified several potential reasons for the limited impact on child outcomes, including late intervention initiation in pregnancy (2nd trimester or later), short intervention duration, and insufficient sample sizes, partly due to loss to follow‐up over time [65]. Additionally, few studies involved relevant stakeholders and reported process evaluations, such as monitoring and assessing whether interventions were delivered as intended, intervention compliance, and participants' acceptability and attitudes toward the intervention [65]. The expert group consulted emphasised that these elements are crucial for ensuring that intervention goals and content are relevant, and the format and compensation are appealing to the participants [65, 67].

Emerging evidence suggests that multi‐behavioural interventions that are initiated during pregnancy and continued into early childhood, targeting parental lifestyle, child feeding practices, and child behaviours, have the potential to be effective in preventing childhood overweight and obesity [57, 62]. Despite a large number of publications and systematic reviews on interventions during the first 3 years of life to address childhood obesity, the evidence on their effectiveness remains limited, as most systematic reviews are based on a limited number of trials. Within EndObesity, an overview of systematic reviews (registered on PROSPERO as CRD42022338940) was conducted, which explored evidence on the effectiveness of early childhood strategies to prevent childhood obesity [68]. We demonstrated that, among the few trials that evaluate single‐behavioural nutritional interventions, only those that reduced early protein intake in infancy through changes in formula composition and limiting consumption of unmodified cows' milk demonstrated positive effects on weight and obesity risk [68, 69, 70, 71]. Evidence on the effectiveness of interventions focused on physical activity or on the weaning process in this age group is limited. Some multi‐behavioural interventions including a physical activity component showed benefits on weight‐related outcomes, whereas all trials that assessed weaning (i.e., baby‐led weaning, nutrition education on weaning practices or timing of introduction of complementary foods) demonstrated no significant results [72, 73, 74, 75, 76, 77]. Yet, for both physical activity and weaning, evidence is not strong enough to draw any definitive conclusions. The absence of clear effects from intervention studies on the timing of complementary feeding contrasts with findings from large cohort studies, which have observed a reduced risk of childhood obesity when complementary foods are introduced after 4 months of age [78]. RCTs assessing the effect of early introduction of complementary foods could be valuable to resolve these discrepancies. Several trials assessed the effect of interventions on responsive feeding (e.g., breastfeeding) and overall; although the quality of the evidence is low and results are inconclusive, it seems that multicomponent interventions may slightly improve weight‐related outcomes [73, 79, 80, 81, 82].

Taken together, interventions during the first 1000 days have shown limited effectiveness in reducing childhood obesity, particularly when initiated late in pregnancy or delivered over a short period. While emerging strategies that extend to early childhood, target multiple behaviours, and incorporate co‐creating principles to address social disadvantage appear more promising, evidence remains sparse, especially for interventions initiated during preconception.

### 1.4 Recommendations From EndObesity for Future Research, Practice, and Policy Development

Over the course of 3 years, extensive multi‐disciplinary collaboration and consensus discussion within EndObesity has led to the development of key recommendations for future research, practice, and policy, as summarised in Tables 1, 2, 3. The following sections explore these recommendations, providing a detailed discussion of the proposed strategies to enhance predictive accuracy of childhood obesity risk, improve intervention effectiveness, and ensure successful implementation in real‐world settings.

**TABLE 1:** Recommendations for research.

*Recommendations for research*
**Prediction strategies**
*Improve predictive models*: Research should focus on improving the predictive accuracy of early life prediction models for childhood obesity using advanced biomarkers, such as omics‐driven data, and advanced modelling approaches like artificial intelligence‐based approaches, while ensuring external validation and generalizability for diverse ethnicities and subgroups and ease of use for healthcare professionals.
*Conduct reviews and meta‐analyses*: Research should include systematic reviews and meta‐analyses focused on the effectiveness and accuracy of childhood obesity prediction models to synthesise current evidence and identify gaps in prediction research.
*Develop and evaluate use of prediction tools for clinical translation*: Research should aim at further development and implementation of prediction tools integrated with prevention strategies and communication tips, followed by effectiveness and process evaluations to determine their impact on communication of risk, sustained behavioural change and risk mitigation.
**Intervention strategies**
*Paternal lifestyle*: Research should specifically explore paternal lifestyle factors and their influence on childhood obesity, as current research predominantly focuses on maternal factors. Examining how paternal involvement and support impacts maternal adherence to healthy lifestyle factors could also provide valuable insights.
*Comprehensive studies on combined lifestyle factors and use of multi‐behavioural interventions*: Research should focus on investigating the combined effects of various parental lifestyle factors (such as BMI, smoking, diet quality, and physical activity) during the first 1000 days on childhood obesity. Understanding, independent, synergistic and cumulative effects can provide deeper insights into preventive strategies. As a next step, multi‐behaviour interventions should be developed, evaluated, and implemented, targeting various parental lifestyle factors simultaneously. Such multi‐behaviour interventions may have greater health benefits for both parents and children compared with single‐behaviour interventions.
*Understanding causality*: Research should include randomised controlled lifestyle intervention trials to gain insights into the causality of observed associations between parental early‐life lifestyle factors and childhood obesity risk. These trials will not only clarify causal pathways but also provide critical evidence on the effectiveness of lifestyle interventions.
*Start time, duration and intensity of interventions*: Research should explore the optimal start time, duration and intensity for preconception and pregnancy lifestyle interventions to maximise their effectiveness in preventing childhood obesity.
*Optimise intervention delivery*: Research should evaluate the most efficient intervention delivery methods focused on health outcomes, cost‐effectiveness, workload for healthcare workers including early‐childhood professionals and social workers, and user engagement, including the use of social media and digital delivery modes (e‐health platforms) to stimulate adherence.
*Evaluate structural components in interventions*: Research should focus on the inclusion and evaluation of structural components in health interventions, such as incentives for healthy food access, service availability, and the impact of urban design.
*Inclusion of social networks*: Research should examine the impact of involving participants social networks in lifestyle interventions, as their inclusion could potentially enhance intervention success.
*Diverse and inclusive strategies*: Research should consider diversity of the participant population, employing culturally sensitive and inclusive outreach strategies and lifestyle interventions.
*Long‐term follow‐up*: Research should ensure long‐term follow‐up of participants in lifestyle intervention studies to track the impact of family lifestyle from preconception through early childhood, and to assess the effects on long‐term offspring development and health outcomes.
*Sample size and dropout rates*: Research should address the issues of sample size and dropout rates in intervention studies to ensure robust and reliable results.
*Large‐scale meta‐analyses and up‐to‐date systematic reviews*: Research should focus on large‐scale meta‐analyses using harmonised data from multiple cohorts to provide more robust and comprehensive evidence on effective prevention strategies for childhood obesity. Regularly updating existing systematic reviews to identify the most effective interventions, will ensure new interventions are based on the latest evidence and best practices for obesity prevention
*Evaluate the impact of protein intake and reduced protein content formulas on childhood obesity*: Research should further explore the relationship between early childhood protein intake, including the use of infant formulas with reduced protein content, and the risk of developing childhood obesity, aiming to determine optimal protein levels for growth and health outcomes and potential lasting benefits of improved feeding strategies in early childhood.
**Implementation strategies**
*Incorporating stakeholder input*: In designing, implementing, and evaluating interventions, researchers should involve key stakeholders, including parents‐to‐be, healthcare professionals including early‐childhood professionals and social workers, public health actors, and industry members using participatory approaches to better adapt programs to family needs and enhance transferability.
*Implementation and evaluation studies*: Conduct implementation and evaluation studies using mixed methods (qualitative and quantitative) to understand why and how interventions work in real‐world settings. These studies should identify barriers and facilitators to successful implementation and scale up, as well as the perceptions of healthcare professionals, and parents, ensuring interventions are effective, sustainable, and adaptable across different context.

**TABLE 2:** Recommendations for practice.

*Recommendations for practice*
**Prediction strategies**
*Implement prediction tools*: Healthcare professionals may benefit from user‐friendly prediction tools to identify infants at high risk of overweight or obesity from the start of life onwards. These toolboxes should include prediction tools, communication tipsheets and targeted prevention strategies.
**Intervention strategies**
*Comprehensive family‐based interventions*: Promote the delivery of multi‐session group educational programs and multi‐level interventions targeting family‐based lifestyle factors from preconception through early childhood in practice. Active involvement of both parents is essential to enhance the effectiveness and stimulate adherence to these interventions.
*Community and family support programs*: Community‐based programs that support families in adopting and maintaining healthy lifestyle practices may be beneficial. These programs could include family cooking classes, peer groups with trained facilitators, group exercise activities and workshops on the importance of a healthy lifestyle during the preconception and pregnancy periods.
*Address structural barriers in health interventions*: Healthcare professionals may benefit from considering and addressing structural barriers such as unemployment, low income, low education levels and disadvantaged environments when designing and implementing health interventions, adapting prevention measures to specific family‐ and social contexts.
**Implementation strategies**
*Early intervention*: It might be beneficial for healthcare professionals to start lifestyle interventions as early as possible during pregnancy or even before conception to maximise their potential impact.
*Training of healthcare professionals*: Ongoing training on the importance of the first 1000 days and childhood obesity prevention is essential to equip healthcare professionals with the knowledge and skills needed to effectively support families and promote long‐term health from the very start of life. Strengthening education and continuing professional development will enhance the capacity of both current and future professionals to address childhood obesity early and effectively.
*Sensitive communication*: Healthcare professionals may benefit from learning how to communicate with families in a sensitive, tailored, non‐judgmental/stigmatising, and engaging manner, focusing on small, achievable steps to promote healthy behaviours. Communication tip sheets may be used to effectively navigate sensitive conversations and ensure comprehensible risk communication with parents.
*Support diverse populations*: Healthcare professionals may consider engaging diverse socio‐economic and ethnic backgrounds through culturally sensitive tools and bilingual professionals, using E‐health platforms for accessible support, and considering home‐visiting for disadvantaged families, to ensure all interventions are designed to be inclusive and supportive.
*Stakeholder collaboration*: Practice guidelines should encourage collaboration between different stakeholders, including researchers, healthcare professionals, educational stakeholders, industry partners, key community figures and parent–child organisations. Such multi‐disciplinary strategies are essential for the successful implementation and evaluation of interventions aimed at preventing childhood obesity.
*Utilise every contact for prevention*: Make every contact count by integrating prevention and intervention opportunities across the life‐course, including antenatal and postnatal care, health contact, childcare services, pre‐schools, schools and parenting programs.

**TABLE 3:** Recommendations for policy.

*Recommendations for policy*
**Prediction strategies**
*Support routine screening and monitoring*: Advocate for policies that encourage routine screening and monitoring of parental health and lifestyle factors during the first 1000 days as part of standard prenatal care.
**Implementation strategies**
*Public health awareness*: European and national policies should increase public awareness of the importance of family‐based healthy lifestyle factors during the first 1000 days.
*Training healthcare professionals*: Organisational policies should allocate resources and finances for training healthcare professionals on the importance of the first 1000 days and preconception health, including multilingual professional development programs.
*Multi‐sectoral collaboration*: European and national policies should develop multi‐sectoral collaboration engaging various sectors, including food, agriculture, transport, housing, employment, leisure industry, education, government and healthcare, to address the obesogenic environment and support healthy family‐based lifestyle factors.
*Integration of expert consultation*: Policymakers should ensure that expert consultation is integrated at multiple stages of research and intervention planning. This consultation can enhance the relevance of research and interventions, ensuring that they address the needs and priorities of various stakeholders, including healthcare providers, researchers and affected populations.
*Funding for early interventions*: Policies should allocate sufficient funding to support early lifestyle interventions that begin during preconception or early pregnancy, and to ensure an appropriate duration of follow‐up. This includes allocating resources for long‐term intervention research—covering the co‐creation phase, implementation, effectiveness and process evaluations and scaling‐up—to build a robust evidence base and support sustainable impact.
*Support access to resources and services*: Advocate for policies that support access to resources and services that facilitate healthy lifestyle choices for families, such as subsidised gym memberships, nutrition counselling and smoking cessation programs.
*Adopt a socio‐ecological perspective*: Policies should adopt a socio‐ecological perspective, recognising the need for structural facilitators such as employment, increased income and enhanced service availability to empower individuals to adopt healthier lifestyles.
*Implement proportionate universalism*: Policies should be based on the concept of proportionate universalism, providing universal access to health services with a scale and intensity proportionate to the level of disadvantage to reduce the social gradient in health and foster social equity.
*Create population‐wide prevention initiatives*: Shift the focus from individual responsibility to societal accountability by creating population‐wide prevention strategies and initiatives that complement individual approaches.

### 1.5 Perspectives for Future Research

Current epidemiological evidence, including novel studies from EndObesity, suggests a strong link between parental lifestyle factors during the first 1000 days and the development of childhood obesity. Yet, there remain important issues to be addressed in future research (Table 1).

First, the consistent associations between parental lifestyle factors during the first 1000 days and childhood obesity risk provide opportunities for early‐life risk prediction. To further enhance the predictive accuracy of prediction models for childhood obesity, future research may focus on incorporating advanced biomarkers in prediction models, such as multi‐omics [83]. Metabolomics, for example, can provide dynamic insights into metabolic processes and potential dysregulation that may not be fully reflected in body composition measures like BMI and body fat mass alone [84, 85, 86, 87]. Within EndObesity, we demonstrated that in a cohort of Dutch school‐aged children, a metabolite profile improved the identification of children with a metabolically unhealthy phenotype, compared with BMI severity only [88]. Additionally, advanced modelling approaches such as artificial intelligence techniques have demonstrated promise in various fields of health research for their ability to handle vast amounts of highly complicated data, and to uncover hidden patterns [89]. Further exploration of these advanced techniques through multi‐cohort analyses integrating diverse datasets from different populations may enhance the generalizability of predictive models across various demographics and environments. Subsequent translation of prediction models into clinical tools, followed by process evaluation, will be essential to assess the impact on sustained behavioural change and risk mitigation, and to understand how they can be effectively implemented in clinical practice.

Second, most evidence on the association between parental lifestyle factors and childhood obesity is derived from observational studies. Observational studies have important limitations, including residual confounding [90]. Randomised controlled lifestyle intervention trials targeting family‐based lifestyle factors throughout the first 1000 days are urgently needed to obtain insights into causality and evaluate effectiveness in preventing childhood obesity. However, designing and conducting such trials pose significant challenges. These include high cost, the need for long‐term follow‐up, substantial risks of participant dropout, and difficulties with collecting funding given the modest long‐term success of previous lifestyle interventions, even though this may well be explained by study limitations. These challenges are especially pronounced in intervention trials starting from preconception onwards, where couples planning pregnancy are often difficult to reach and may not yet be engaged with healthcare services. Additionally, not all enrolled couples will go on to conceive or develop the outcome of interest, requiring large initial sample sizes. Despite these challenges, several recent RCTs from preconception onwards demonstrate that such studies are possible. The Generation R Next Intervention Study (‘Growing Up Optimally’) is one such example. This is a novel ongoing population‐based intervention study for couples from preconception onwards where close collaboration with the municipality, healthcare workers, local stakeholders, and input from parents‐to‐be at several design stages facilitated effective recruitment, enrolment, and retention. Similarly, the Norwegian ‘Before the Beginning Trial’ identified women of reproductive age through the National Population Register and leveraged social media to boost compliance and maintain adherence [91]. Strong international collaboration is needed to build on these experiences, sharing best practices for the design and management of RCTs from preconception onwards, enabling harmonised data collection, and supporting future large‐scale meta‐analyses aimed at identifying consistent and novel strategies for childhood obesity prevention. To further improve intervention success, our scoping reviews and consortium discussions identified key areas for future research. These include the development of multi‐behavioural intervention programs, adherence to theoretical frameworks, involvement of women's partners or relatives, identification of critical periods for intervention initiation, determining optimal delivery methods, and establishing the right duration and intensity of interventions to promote sustained behaviour change and improved health outcomes [65]. Carefully considering sample size, dropout rates, and follow‐up duration in intervention studies is essential to reliably assess their long‐term health effects. Our consortium strongly recommends involving key stakeholders such as expectant parents, public health actors, and healthcare professionals, including social workers and early‐childhood professionals, in co‐creating and implementing interventions to ensure that designed programs are not only scientifically sound but also resonate with the needs and experiences of the target population. To strengthen implementation efforts, European‐level collaborations should prioritise the exchange of practical strategies to enhance participant compliance, inclusion, and long‐term follow‐up. These strategies may include the use of social media, not only to share information but also to foster peer support through forums or closed groups that help sustain engagement, the establishment of parent–child panels to better understand what families need to achieve compliance with interventions and to remain willing to participate in follow‐up studies, and the integration of home visits to build trust and reduce barriers to participation. These types of participant‐centred approaches are essential for improving real‐world effectiveness and scalability of early‐life interventions.

In addition, given the challenges of conventional RCTs, we recommend incorporating alternative and complementary strategies. These include trial simulation studies using existing cohort data, and analyses of real‐world data after the implementation of public health measures (such as sugar taxes or anti‐smoking policies) which can provide valuable causal insights and insights into the cost‐effectiveness of these public campaigns. International comparisons of countries with varying preventative policies and large‐scale meta‐analyses can also help identify effective strategies and improve generalizability. Embedding studies within existing infrastructures and fostering collaboration with local authorities can further support the feasibility, scalability, and sustainability of early prevention efforts. Such an inclusive and participatory approach has been shown to enhance the design and implementation of effective prevention strategies [62, 92]. An example embedded within EndObesity is the French prEgnanCy and eArly Childhood nutrItion triaL (ECAIL, ECAIL; Clinicaltrials.gov NCT03003117) [93], which aims to assess the effectiveness of the multi‐behavioural and multilevel Malin program. ECAIL is a ‘participatory action research’ project, co‐created with members of the Malin nonprofit association and its stakeholders, including the French Red Cross and two French paediatric societies. This program, which begins during pregnancy and spans the first 1000 days, operates at multiple levels of the socio‐ecological model, both individual and structural [57, 94]. Grounded within the framework of the social cognitive theory [95], the first component seeks to build knowledge, skills, self‐efficacy, and social support regarding feeding practices and lifestyle behaviours, including practical advice on responsive feeding (e.g., breastfeeding), a balanced diet (e.g., multi‐cultural recipes), and guidance on maintaining an active lifestyle (e.g., limiting screen exposure) [95]. A major principle of the Malin program is the setting of individualised, gradual, and achievable goals. At a more upstream level, the second component adopts a non‐stigmatising approach to improve the availability, accessibility, and affordability of healthy foods. Its main goal is to alleviate the financial burden of healthy eating on family budgets and reduce the trade‐offs that contribute to food insecurity. Parents are given the opportunity to subscribe to community‐supported agriculture baskets of fresh, organic fruits and vegetables at a significantly reduced cost from the third trimester of pregnancy to the child's second birthday. To promote homemade meals, parents also have access to triannual online sales that allow them to purchase cooking equipment and kitchen utensils at reduced prices (from pots and saucepans to food processors). Finally, discount vouchers to access foods recommended by the National Nutrition and Health Program are sent to the families' homes when the child is 6, 9, 12, 16, and 20 months old, and vouchers can be used in all supermarkets. Malin was developed at the cutting edge of social innovation, health promotion, and solidarity, in close collaboration with social, health, and early childhood stakeholders as well as recipients (i.e., parents). This partnership was essential to reach and follow up vulnerable and hard‐to‐reach families. Implementation and process evaluation studies using mixed methods will help understand why or why not, how, for whom, and in which contexts interventions work in real‐world settings. These studies may help identify barriers and facilitators to successful implementation and ensure that interventions are effective, sustainable, and adaptable across different contexts.

Third, the strongest childhood obesity prevention effects to date have been achieved through optimised infant feeding strategies, particularly by limiting milk protein intake in infancy. These findings highlight the need for further research on the potential lasting benefits of improved feeding strategies in early childhood, including modifying both the quality and amount of protein provided or exploring the potential of improved feeding strategies in toddlerhood [96, 97].

Finally, it is important to consider potential biases that may have influenced findings within the EndObesity Consortium. Most available data came from a selection of European countries, particularly the Netherlands, Germany, France, Ireland, and Italy, while data from Eastern, Northern, and parts of Southern Europe were underrepresented. Cultural practices, dietary patterns, socio‐economic circumstances, and healthcare systems differ substantially across Europe, potentially influencing both the determinants of childhood obesity and the effectiveness of prevention strategies. In addition, some topics for childhood obesity prevention remained underexplored in our consortium, such as cost‐effectiveness and economic evaluations due to time and financial constraints. By expanding research to more diverse populations and exploring these underrepresented topics, future studies can further increase the generalizability and applicability of our findings.

### 1.6 Recommendations for Practice

As part of a systems approach, healthcare professionals, including early‐childhood professionals and social workers, can play a crucial role in the prevention of childhood obesity in the first 1000 days (Table 2).

First, to identify those families that will gain most from early‐life obesity prevention strategies, healthcare professionals may benefit from using prediction tools. Yet, the implementation of prediction tools into clinical practice poses challenges [98, 99]. EndObesity explored healthcare professionals' perceptions regarding the use of prediction tools in clinical practice, revealing general positivity but also highlighting potential negative connotations and ethical issues, such as perceived stigmatisation and parental guilt with being labelled high‐risk [100]. According to healthcare professionals, prediction tools should be simple, user‐friendly, and require minimal actions, using readily available data, to maximise successful use in clinical practice. As a consequence, when incorporating omics or genetic data into prediction models, a balance needs to be found between adding complexity and maintaining usability. To enhance awareness of childhood obesity prevention in early life and educate healthcare professionals, researchers, and students, EndObesity developed an online accredited e‐learning module in close collaboration with the Child and Family Health Academy. This module is available worldwide and focusses on the early‐life multifactorial origins of childhood obesity, prevention potential, facilitators and barriers, and implementation of childhood obesity prediction tools [101]. By supporting education and continuing professional development, the module aims to strengthen the capacity of current and future professionals to address childhood obesity from the earliest stages of life.

Second, supporting multi‐session educational programs and interventions in clinical practice is essential to promote family‐based lifestyle factors during the first 1000 days. These programs should actively involve both parents to enhance their effectiveness. Within EndObesity, we demonstrated that attending antenatal parenting preparation sessions was associated with healthier parental lifestyles during pregnancy [44]. Community‐based initiatives, such as family cooking classes, peer groups with trained facilitators, group exercise activities, and workshops, can further support families in adopting and maintaining healthy behaviours. Such community‐based interventions provide an opportunity for resources within the community to be drawn upon, with many opportunities to partner, collaborate, and enrich initiatives [102]. These community‐based and community‐wide multi‐strategy approaches to obesity prevention have proven effective in improving health [10, 102, 103, 104, 105]. To enhance the impact of these clinical interventions and community programs, healthcare professionals should have dedicated time during family visits to address potential structural barriers faced by families with lower socio‐economic positions, such as unemployment, low income, limited education, and disadvantaged environments. Applying the framework of proportionate universalism ensures that interventions are tailored to the specific needs of these families, making them more equitable and effective [107]. Given the time constraints healthcare professionals often face, it is crucial to integrate these efforts into routine care. Practical strategies may include integrating screening tools to identify social needs, such as the WE CARE survey and PREPARE screening tool [107, 108], incorporating referral systems into electronic health records to connect families directly with local services such as food banks, housing support, or debt counselling, and fostering active collaboration with social workers, youth health professionals, or interdisciplinary care teams. Expanding prevention efforts across the life course, through antenatal and postnatal care, preschools, and parenting programs, enhances reach and effectiveness. Multidisciplinary teams should deliver these interventions across multiple settings, such as home visits, which have proven effective in engaging socially disadvantaged families, ensuring every contact point with families serves as an opportunity for prevention [62].

Third, as some healthcare professionals experience discomfort when talking about weight and risk prediction with parents due to the sensitivity and stigma surrounding these topics, healthcare professionals will likely benefit from training on how to communicate about improving lifestyle and obesity prevention in a sensitive, tailored, non‐judgmental/stigmatising, and engaging manner [57, 109, 110]. Communication tip‐sheets on discussing obesity prevention and a healthy lifestyle with parents and families have been developed within EndObesity and can assist healthcare professionals in navigating difficult conversations and ensuring comprehensible risk communication [99]. As healthcare professionals encounter families with diverse educational and cultural backgrounds, the use of culturally sensitive, multilingual tools ensures that interventions are inclusive and accessible to all families.

Finally, several evidence‐based interventions are ready for implementation in clinical practice to support healthy growth and obesity prevention. A multi‐behavioural approach that promotes responsive feeding, healthy dietary intake, and an active lifestyle across all family members is key. This includes encouraging breastfeeding when possible, as well as the use of infant formula with lower protein content, more similar to human milk, when breastfeeding is not possible [69]. Additionally, unmodified cow's milk should not be introduced as a drink during infancy, and its intake in toddlers should be moderated, as observational studies suggest an association with later obesity [69, 70, 71]. Incorporating these recommendations into routine care can help establish healthier early‐life nutrition practices.

### 1.7 Recommendations for Policy

Effective policy is essential to create an environment that supports and encourages families in the prevention of childhood obesity (Table 3).

First, integrating routine screening and monitoring of parental health and lifestyle factors into standard prenatal care can be beneficial. To make this feasible, structural policy changes should ensure healthcare professionals have the necessary time and resources available. Several successful models illustrate how this integration can be achieved in practice. For example, the Centring Pregnancy model combines group‐based prenatal care with structured health education and peer support, and has been associated with improved maternal behaviours and birth outcomes [112]. The UK's national Healthy Start scheme provides low‐income pregnant women and families with young children with financial support to buy fruits, vegetables, and milk, as well as free vitamin supplements, helping reduce nutritional inequalities and promote healthy choices [113]. However, to drive widespread adoption, such strategies must also be supported by evidence of cost‐effectiveness. Evidence from economic research, such as the work of James Heckman, has already shown that investing in early‐life interventions yields high returns in terms of long‐term health, social, and economic outcomes [114]. To further support widespread implementation, there is a need for robust health economic evaluations demonstrating the cost‐effectiveness of such approaches in real‐world settings.

Second, European and national policy should prioritise raising public awareness about the importance of a healthy lifestyle during the first 1000 days, as early education and awareness are key to preventing childhood obesity. To support this, long‐term funding is needed for implementing early‐life interventions, the training of healthcare professionals, social workers, and early childhood professionals about the significance of the first 1000 days and preconception health, and to support access to resources and services that facilitate healthy lifestyle choices for families. These might include subsidised gym memberships, incentives to access healthy foods, nutritional counselling, and smoking cessation programs. By ensuring structural facilitators, such as stable employment, increased income, and improved access to healthcare, nutrition, and social support services, policies can empower individuals to adopt and sustain healthier lifestyles. Shifting the focus from individual responsibility to societal accountability through population‐wide prevention and health promotion initiatives is critical to shifting the distribution of risk factors at the population level. Multi‐sectoral collaboration across the food industry, agriculture, education, and transportation can help reduce obesogenic environments, making healthy living more accessible and sustainable for all. Several successful worldwide examples have shown how public policy can shift population health outcomes. For example, government‐imposed taxes on sugar‐sweetened beverages in countries such as Mexico and the United Kingdom have resulted in widespread product reformulation and significant reductions in consumption [115]. Tobacco control policies, including smoking bans and the implementation of a ‘smoke‐free generation’ strategy in the Netherlands, have dramatically lowered smoking rates and changed social norms [116]. The mandatory fortification of food products with folic acid in the United States and other countries has led to a marked decline in neural tube defects such as spina bifida [117, 118]. These examples demonstrate how regulatory and fiscal policies can reshape industry behaviour and protect public health. Future efforts could include stronger restrictions on advertising unhealthy foods to children, clearer front‐of‐pack labelling, and tighter regulation of food composition. Only by transforming the environments in which families live, learn, and work can we create lasting change and turn the tide on childhood obesity.

## 2 Conclusion

The first 1000 days of life represent a unique opportunity to prevent childhood obesity from the start of life onwards. Despite its tremendous potential, prevention strategies have thus far led to disappointing results. Findings from the EndObesity Consortium highlight the importance of collaborative action across clinical, community, and policy levels to reduce health disparities and obesity risk in future generations. Translating robust evidence from observational studies on parental and early‐childhood family‐based lifestyle factors in the first 1000 days, alongside findings from randomised trials on optimised infant feeding strategies, into multi‐behavioural interventions is essential. A multi‐faceted approach is needed, combining individualised interventions with supportive policies that promote routine risk screening, increase public awareness, and create environments that facilitate a healthy lifestyle from the earliest stages of life onwards to break the intergenerational obesity cycle.

## Author Contributions

M.S. and R.G. were primarily responsible for the conception, drafting, and writing of the manuscript. All other authors contributed by critically reviewing the manuscript for intellectual content and approved the final version for submission.

## Conflicts of Interest

The IISPV group conducts research partially funded by industry (Nestlé, Südzucker group, DGC cooperative) not related to the purpose of this review.

## Supporting information


**Data S1:** Supporting Information.

## Data Availability

The data that support the findings of this study are available on request from the corresponding author. The data are not publicly available due to privacy or ethical restrictions.
