# Supplementary material for: First 1000 Days Strategies to Prevent Childhood Obesity: A Narrative Review and Recommendations From the EndObesity Consortium
Source: Pediatr Obes. 2025 Oct 17;21(1):e70060. doi: 10.1111/ijpo.70060 (PMC12696515; doi:10.1111/ijpo.70060)
Supplement: Supplementary file 1 — Data S1: Supporting Information. [file IJPO-21-e70060-s001.pdf]

Supplementary Materials

First 1,000 days strategies to prevent childhood obesity: a narrative review and recommendations from the EndObesity Consortium

Running title: Childhood obesity prevention in the first 1,000 days

Mireille C. Schipper[a,b], Anna Manshanden[c], Kaat Philippe[d], Natàlia Ferré[e,f], Veronica Luque[e,f] , Marion Lecorguillé[g], Adrien M. Aubert[d], Kathrin Guerlich[h,i], Camille Le Gal[g], Shweta Feher[h], Ester Parada-Ricart[e,f], Joaquin Escribano[e,f], Veit Grote[h,i] , Berthold Koletzko[h,i,j], Teresa Primavesi-Poggio[k], Katharina Reiss[l], Jaap C. Seidell[c] , Sandrine Lioret[g], Catherine M Phillips[d], Barbara Heude[g] , Romy Gaillard[a,b]

[a]The Generation R Study Group, Erasmus MC, University Medical Center, Rotterdam, the Netherlands

[b]Department of Pediatrics, Sophia's Children's Hospital, Erasmus MC, University Medical Center, Rotterdam, the Netherlands

[c]Department of Health Sciences, Faculty of Science, Vrije Universiteit Amsterdam, Amsterdam, Netherlands

[d]School of Public Health, Physiotherapy and Sports Science, University College Dublin, Dublin, Ireland

[e]Pediatrics Nutrition and Development Research Unit, Institut d'Investigació Sanitària Pere Virgili, Reus, Spain

[f]Universitat Rovira i Virgili, Reus, Spain

[g]Université Paris Cité and Université Sorbonne Paris Nord, Inserm, INRAE, Center for Research in Epidemiology and StatisticS (CRESS), Paris, France

[h]Division of Metabolic and Nutritional Medicine, Department of Pediatrics, Dr. von Hauner Children's Hospital, LMU University Hospital, LMU Munich, Germany

[i]German Center for Child and Adolescent Health, site Munich, Germany

[j]Child Health Foundation - Stiftung Kindergesundheit, c/o Dr. von Hauner Children's Hospital, Munich, Germany

[K]European Foundation for the Care of Newborn Infants, Munich, Germany

[l]Healthy Start Network, Federal Office for Agriculture and Food, Bonn, Germany

Corresponding Author: Romy Gaillard, The Generation R Study Group, Erasmus University Medical Center, PO Box 2040, 3000 CA Rotterdam, the Netherlands. E-mail: r.gaillard@erasmusmc.nl. Telephone number: 010 – 7043405.

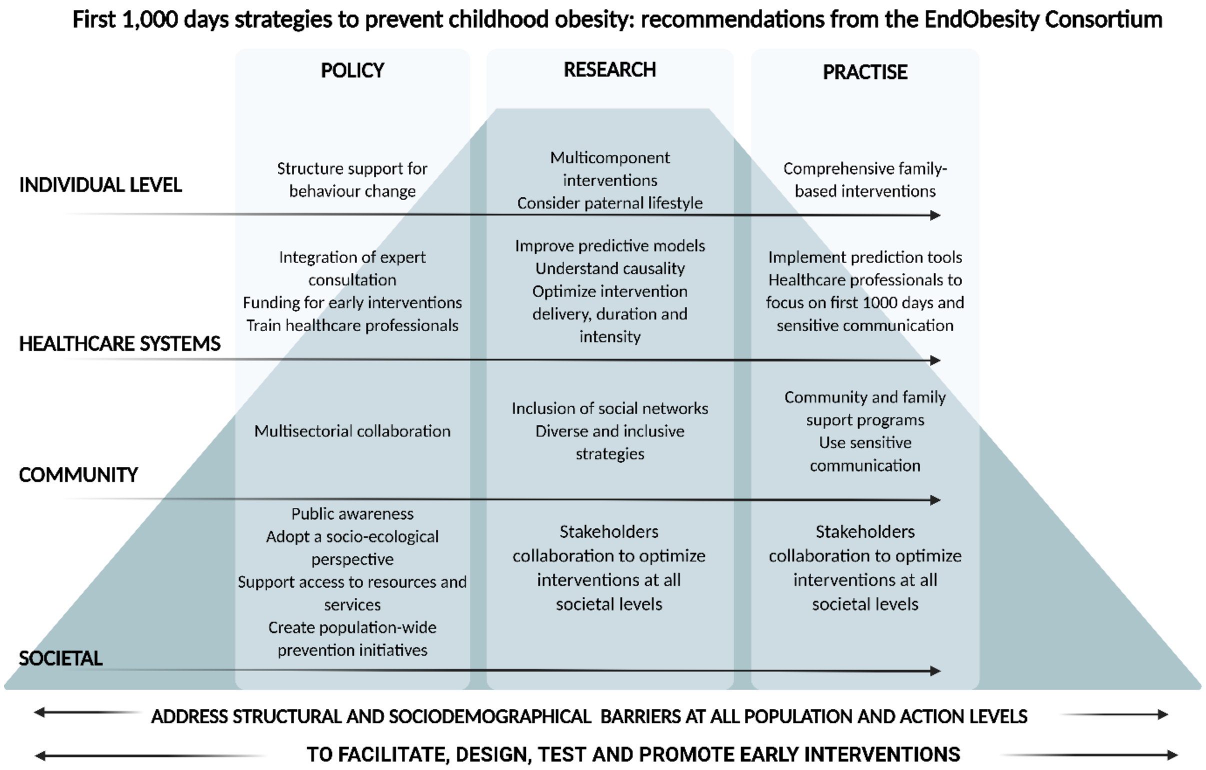

Figure S1. Schematic overview of the recommendations of the EndObesity Consortium to prevent childhood obesity in the first 1,000 days of life. This figure is intended for use in dissemination activities to clearly communicate key recommendations to researchers, policymakers, healthcare professionals, and other stakeholders.[1]

1. Created in BioRender. Luque, V. (2025).
